# Identification of rare variants of allergic rhinitis based on whole genome sequencing and gene expression profiling: A preliminary investigation in four families

**DOI:** 10.1016/j.waojou.2019.100038

**Published:** 2019-06-13

**Authors:** Yuan Zhang, Jingyun Li, Yali Zhao, Chengshuo Wang, Luo Zhang

**Affiliations:** a Department of Otolaryngology Head and Neck Surgery, Beijing TongRen Hospital, Capital Medical University, Beijing 100730, PR China; b Beijing Key Laboratory of nasal Diseases, Beijing Institute of Otolaryngology, Beijing 100005, PR China; c Department of Allergy, Beijing TongRen Hospital, Capital Medical University, Beijing 100730, PR China

**Keywords:** Allergic rhinitis, Whole genome sequencing, Gene expression profiling, FLT1, VEGFB, ITGA2, Rare variants

## Abstract

**Background:**

Despite the success of genome-wide association studies for allergic rhinitis (AR), no definitive causal variants have been identified, and a substantial portion of the heritability of the disease is yet to be discovered.

**Methods:**

Four families, each with at least 1 parent and one child suffering from dust mite (DM) AR, were recruited, and whole-genome sequencing was performed on samples from 9 eligible individuals from these families. Conjoint analysis was performed for existing gene expression profiling data in the literature and the whole genome sequencing data obtained for these individuals; for presence of family-specific variants segregating with AR and the pathways involved. Similar analyses were also performed with data obtained for 96 sporadic house dust mite (HDM) AR patients and 96 healthy controls.

**Results:**

Three rare variants in three genes (FLT1_c.603A ​> ​T; VEGFB_c.322A ​> ​C; and ITGA2_c.502+1G ​> ​A), which are involved in Focal Adhesion pathway, were identified in affected, but not unaffected, subjects in two families. VEGFB_c.322A ​> ​C and/or ITGA2_c.502+1G ​> ​A were further detected in all DM AR patients but not in any healthy individuals in 1 family; which was further investigated for members. The 3 identified variants were not found in any of the sporadic DM AR patients or healthy controls.

**Conclusion:**

Despite the relatively small sample size, this study has identified several potentially functional rare variants in AR candidate genes, and it provides a platform for future work in larger numbers of families and sporadic individuals for a better understanding of the genetic basis of AR.

## List of abbreviations

AR: allergic rhinitis
BCAP: B cell adaptor for phosphatidylinositol 3-kinase
C11orf30: chromosome 11 open reading frame 30
DEG: differentially expressed gene
DM: dust mite
FLT1: fms related tyrosine kinase 4
GWAS: genome wide association study
HLA-DRB4: major histocompatibility complex, class II, DR beta 4
IgE: immunoglobulin E
ITGA2: integrin subunit alpha 2
MRPL4: mitochondrial ribosomal protein L4
NEC: nasal epithelia cell
NP: nasal polyp
SNV: single nucleotide variants
VEGFB: vascular endothelial growth factor B

## Introduction

Allergic rhinitis (AR) is an inflammatory disease of the nasal mucosa, which is induced by an immunoglobulin E (IgE)-mediated reaction in allergen-sensitized subjects, and it has increased in prevalence over the last decade.^1^, ^2^, ^3^, ^4^ Epidemiological studies have revealed that the prevalence of AR has increased progressively in the more-developed countries, and it currently affects up to 40% of the population worldwide.^2^, ^3^, ^5^, ^6^ The global rising trend of AR has also been observed in the Asia Pacific region^7^ and China in the past two decades.^8^ A more recent study investigating the prevalence of AR in 18 major cities in China had indicated that there was an overall increase of 6.5% in the prevalence of self-reported AR during a 6-year period (from 11.1% in 2005 to 17.6% in 2011) in the general Chinese adult population.^9^ Although AR is not life threatening, AR has a serious negative influence on the lower airway,^10^, ^11^ the patient's quality of life and psychological status,^12^, ^13^, ^14^, ^15^, ^16^, ^17^ and it imposes a huge socioeconomic burden.^18^, ^19^

Allergic diseases, including AR, are complex genetic diseases resulting from the effect of both multiple genetic and interacting environmental factors on their pathophysiology.^20^ Although there has been a tremendous surge in our understanding of the genetics of allergic disease, particularly asthma,^21^ with the advance of genome-wide linkage studies, candidate-gene association studies and genome wide association studies (GWASs), relatively little is known of the genetics of AR. To date only one GWAS of AR has been conducted in a Singaporean Chinese population, and it reported that mitochondrial ribosomal protein L4 (MRPL4) and B cell adaptor for phosphatidylinositol 3-kinase (BCAP) were two novel candidate genes for atopy and AR.^22^ More recently, a meta-analysis of GWASs conducted among 4 large European adult cohorts has identified major histocompatibility complex, class II, DR beta 4 (HLA-DRB4), chromosome 11 open reading frame 30 (C11orf30) and leucine rich repeat containing 32 (LRRC32) loci to be significantly associated to either AR phenotype or grass sensitization.^23^ However, it has increasingly been recognized that most of the variants identified by GWASs so far confer relatively small increments in risk and explain only a small proportion of familial clustering. This also indicates a major gap in profiling the genetics of allergic disease, i.e. “missing” heritability.^24^ Many explanations for this missing heritability have been suggested^25^; foremost among them is that GWAS is reliant on genotyping of common haplotype blocks and therefore is restricted in its ability to detect rare risk alleles that might be contributing to the disease.^25^ Another explanation for the lack of functional variant identification is that the degree of genetic heterogeneity for common diseases is markedly higher than previously thought, possibly due to the presence of rare or even family-specific mutations with a large effect.^26^ We have hypothesized that individual families segregate “family-specific” variants or pathways contributing to AR susceptibility and that at least one family-specific variant in the same pathway is necessary for disease development within individuals in the family. To test this hypothesis, we have investigated family-specific variants segregating with AR in 4 families identified with 1 child and at least 1 of their patients suffering from dust mite (DM)-induced AR, using whole-genome sequencing analysis combined with existing gene expression profiling data.

## Methods

### Study population

Four AR families and 96 AR sporadic subjects suffering from DM-induced AR, as well as 96 healthy controls, were recruited from the outpatient clinic of the Allergy Department in Beijing TongRen Hospital, during the period of February 2012 to February 2016.

All subjects had a history of AR for at least 1 year and fulfilled all criteria of the Allergic Rhinitis and its Impact on Asthma (ARIA) guidelines^27^; including: i) presence of persistent or discontinuous symptoms of anterior rhinorrhoea, continuous sneezing, nasal obstruction and itching, ii) demonstration of a pale and oedematous nasal mucosa, nasal discharge and swollen inferior turbinates by nasal endoscopy, and iii) positive serum antigen-specific IgE, measured by the ImmunoCAP 250 system (Pharmacia, Uppsala, Sweden). A diagnosis of AR was further confirmed by the presence of symptoms induced by exposure to an allergen shown to produce a strong positive skin test response. Serological testing was performed by specialist technicians, while the diagnosis for AR was made by clinical rhinologists. The 96 sporadic DM AR subjects were also shown to have a family history of allergic disease.

The tested antigens included DM (Der f and Der p); seasonal grass pollens (Giant Ragweed; Mugwort; Lamb's quarters; Humulus; *Chenopodium album*); animal hair (dog and cat); molds (indoor and outdoor mustiness or floricultural environment) and cockroach. The serum antigen-specific IgE Phadiatop™test was also performed to test for sensitization to common allergens, and subjects were considered to be sensitized to the allergens when the serum IgE was ≥0.35 kU/l. The CAP classification system divides results into 7 categories from 0 to 6; which are scored as follows: class 1: 0.35–0.70 kU/l; class 2: 0.71–3.5 kU/l; class 3: 3.51–7.5 kU/l; class 4: 7.6–17.5 kU/l; class 5: 17.6–50 kU/l, and class 6: 〉50 kU/l. This unit reported by CAP was in accordance with the defined WHO serum standard IRP75/520. Procedural recommendations for total IgE (Pharmacia, Uppsala, Sweden) were followed strictly, and the results were considered positive if a value for total IgE was>100 kU/l.^28^

Only subjects sensitized to DM and with a confirmed diagnosis of AR were included in the present study. For the present study, individuals were considered to be sensitive to DM allergens if the levels of Der f and Der p sIgE were equal to or above class 2.

AR subjects were excluded if they had i) co-morbid asthma, eczema, or any other allergic disease; ii) hypertension, diabetes or other chronic diseases; or iii) tumor in the nasal cavity or any other inflammatory nasal disease such as sinusitis and nasal polyps. The diagnosis of asthma was confirmed by a chest physician according to the Global Initiative for Asthma (GINA) guidelines.^29^

A total of 96 healthy adult volunteers were also recruited as controls from an ethnically similar local population, to determine background allele frequencies in the population. These control subjects presented no clinical features, local nasal cavity signs or a family history of allergic disease, and they showed negative serum antigen-specific IgE by Phadiatop™testing.

All subjects were of Han Chinese ethnic origin from the Beijing region, China, and they provided written informed consent prior to entry in the study. The study protocol was approved by the Ethics Committee of Beijing TongRen Hospital and performed in accordance with the guidelines of the World Medical Association's Declaration of Helsinki.

### Whole genome sequencing and bioinformatics analysis

The genomic DNA was assessed in samples from nine individuals from the four families affected by AR, using whole genome sequencing. Library preparation and genome capture were done with Illumina TruSeq DNA Sample Preparation Kit (Illumina, USA), and whole genome sequencing was performed on illumina HiSeq2000 platform (Illumina, USA). Initial alignment was performed with Burrows-Wheeler Aligner (BWA) according to the reference human genome build hg19. single nucleotide variants (SNVs), and insertions/deletions (Indels) were determined using the Genome Analysis Toolkit (GATK). Annotation of all variants was performed using SnpEff.

Variants were filtered and low quality variants were excluded using the following criteria: 3 or more variants detected within 10 bp; coverage depth less than 5 folds; mapping quality score less than 30; genotype quality less than 30.

Taking into consideration the likely pathogenicity, we also filtered out heterozygous variants (nonsynonymous, splice site acceptor or donor variants and Indels), which were detected in both the affected parent and the child and were absent from dbSNP143 and 1000 Genomes Project.

The pathogenicity risk of nonsynonymous variants was estimated using Sort Intolerant from Tolerant (SIFT), Polymorphism Phenotyping v2 (PolyPhen2), PhyloP and mutation taster. The prediction of new splice site was tested with four different tools: namely, Human Splicing Finder, MaxEntScan, Spliceman and NetGene2. Potentially deleterious variants were reviewed in the Exome Aggregation Consortium (ExAC).

Three-dimensional (3D) modelling of wild type and missense variants was performed using the SWISS-MODEL. Data obtained by the homology models were visualized using Swiss-PdbViewer 4.1.

### Global gene expression profiling access

Data from a previous global gene expression study of nasal mucosa from 7 AR patients and 5 non-allergic control subjects (GSE43523) were used to analyze the distinct expression gene shared with whole genome sequencing data. Gene Ontology (GO) function enrichment was generated by DAVID Bioinformatics Resources. KEGG and Reactome pathway databases were used to obtain canonical pathway and disease and bio functions. Values of *P* ​< ​0.05 were considered to be statistically significant.

### Mutation validation

After filtering against multiple databases, Sanger sequencing was used to confirm that mutations were more likely to be responsible for AR. Direct polymerase chain reaction (PCR) products were sequenced using Bigdye terminator v3.1 cycle sequencing kits (Applied Biosystems, Foster City, CA) and analyzed using the ABI 3700XL Genetic Analyzer.

## Results

Fig. 1 and Supplementary E1 show the clinical and demographic data of the 9 affected members in 4 families assessed for causative AR genes using whole genome sequencing. An average of 51.8 ​Gb of sequence was generated from DNA samples from each of the affected individual, and demonstrated that on average, 91.7% of bases mapped to the reference genome with a mean coverage of 15× (Supplementary E2). Genome sequencing analysis demonstrated mean values of 3,323,100 SNVs and 408,518 Indels in the samples. We focused primarily on nonsynonymous variants, splice site acceptor or donor variants and coding Indels (NS/SS/I) that were most likely to be pathogenic and found that after filtering outshared variants found in dbSNP143 and 1000 Genome Project, a total of 141–179 NS/SS/I variants were detected per affected individual (Table 1). Considering that candidate variants would be co-segregated between the affected parent and the child in each family, assessment of genes with damaging alleles found in at least 2 families demonstrated 3 genes (KCNG4, NCOA6 and KIAA1217) that contain five heterozygous missense mutations (Table 1 and Supplementary E3). However, we further searched the previous relevant studies and participation in biologic categories strongly implicated in AR, there was no evidence that these 3 genes were associated with AR or atopic genotypes.

**Fig. 1:**
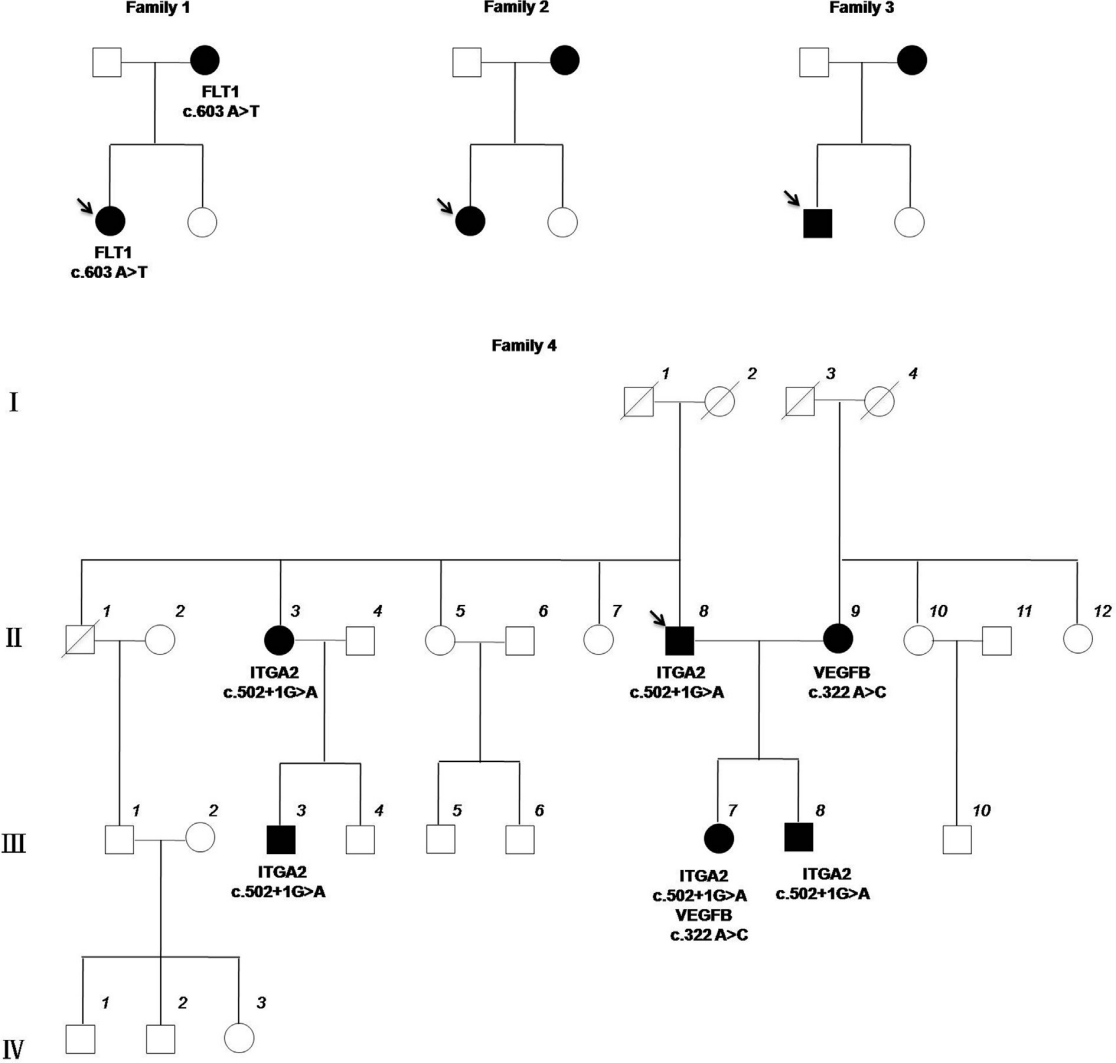
Pedigrees of 4 families with HDM AR. Filled-in symbols indicate individuals with HDM AR, empty circles indicate unaffected individuals, and symbols with a slash through them indicate deceased individuals. Arrow indicates the probands of families. The mutation present in each family is indicated below the corresponding affected individuals.

**Table 1:** Identification of the causal gene by whole genome sequencing.

Filtering	Family1		Family2		Family3		Family4		
	MotherChild		MotherChild		Mother Child		Mother Father Child1		
NS/SS/I^a^	141	165	133	142	143	142	179	152	163
Parent-child shared NS/SS/I	78		72		64		147		
Family shared NS/SS/I (shared by at least two families)	2		1		1		2		
	KCNG4, NCOA6		KIAA1217		KIAA1217		KCNG4, NCOA6		

Variants were excluded in dbSNP143, 1000 Genomes.

a NS, nonsynonymous variant; SS, splice-site acceptor or donor variants; I, coding insertions or deletions.

**Fig. 2:**
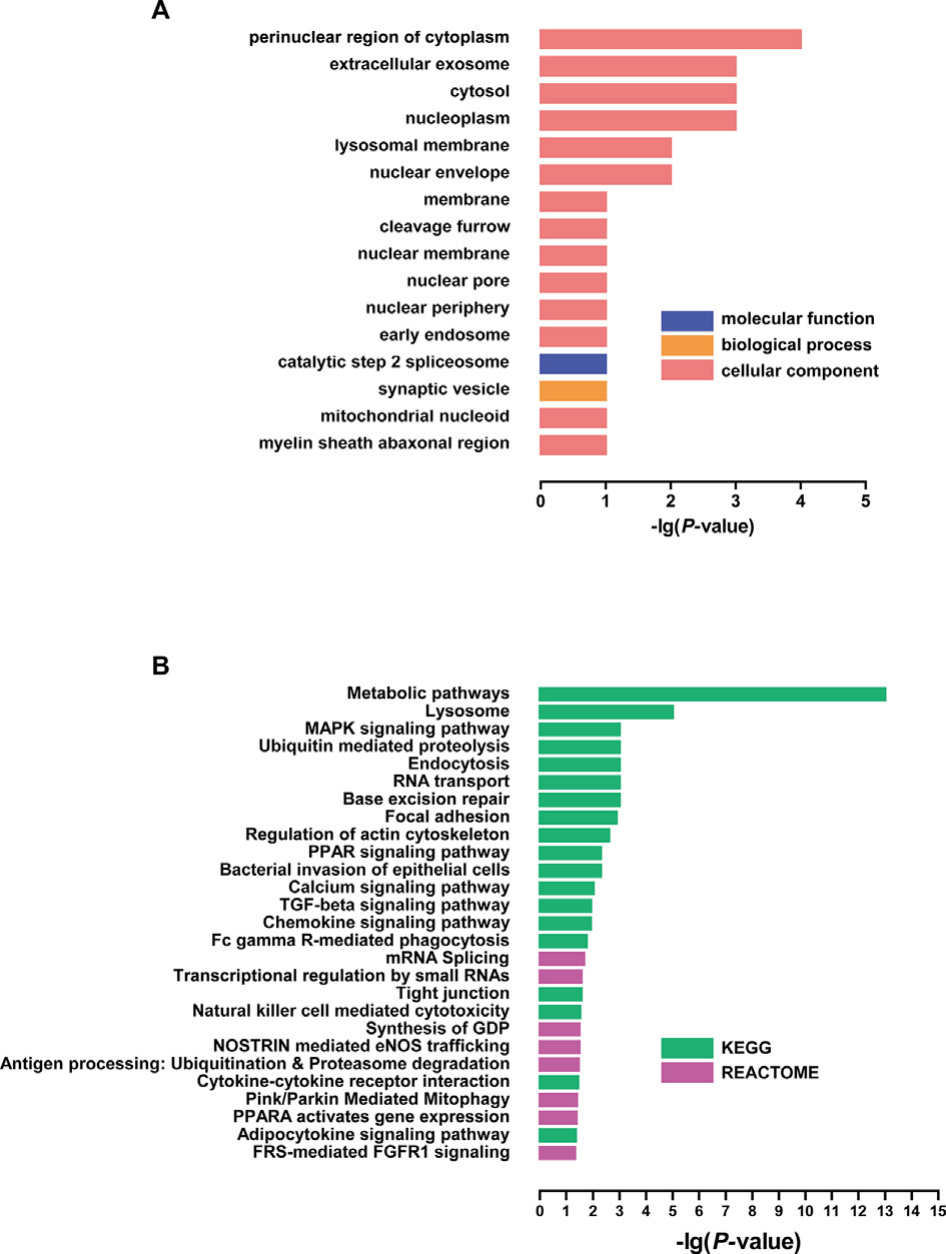
Gene ontology enrichment (A) and pathway analysis (B) performed by DAVID for gene expression profiling (GSE43523) from the GEO database.

**Fig. 3:**
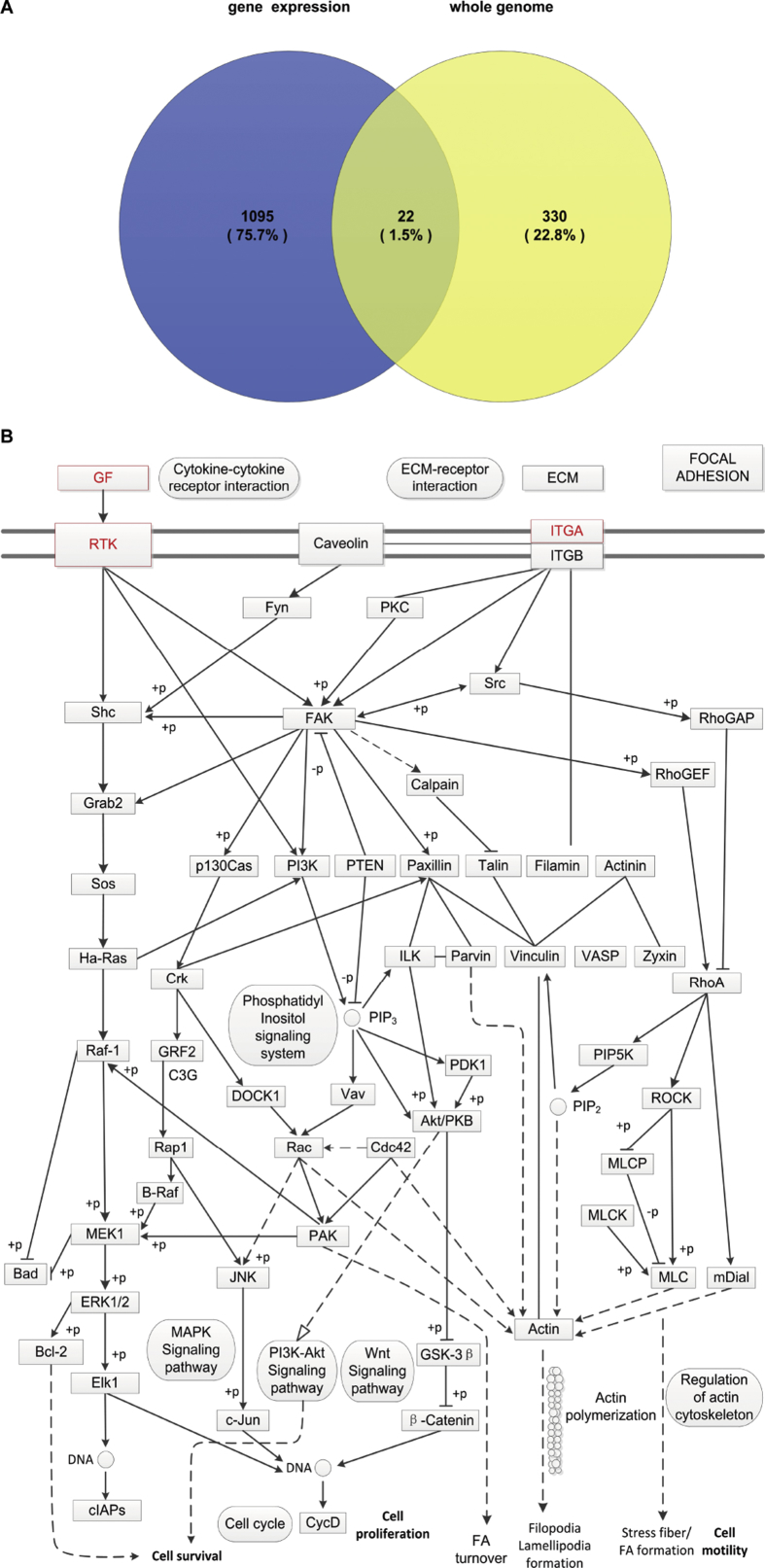
A.Venn diagram of shared genes between distinct gene expression profiling and whole genome sequencing data. B. KEGG pathway for local adhesion. The significant shared genes between distinct gene expression profiling and whole genome sequencing data are shown in red.

As AR is recognized to be genetically heterogeneous, we performed an integrated analysis across the whole genome sequencing data obtained in the present study and data from 1 previous gene expression profiling study of AR (GSE43523) to identify “family-specific” rare variants or pathways in at least 2 families. Microarray data for nasal mucosa from 7AR patients and 5 non-allergic control subjects enrolled in the present study demonstrated a total of 1117 differentially expressed genes (DEGs) between the two groups of subjects. These DEGs were loaded into DAVID for GO enrichment analysis, employing all significantly enriched GO terms including categories of biological process, molecular function and cellular component (Fig. 2A). Pathway analysis showed a total of 27 pathways enriched significantly (Fig. 2B). Furthermore, the 2 sets of candidate genes of whole genome sequencing and DEGs of gene expression profiling shared 22 genes (Fig. 3A); of which 3 (Integrin Subunit Alpha 2 (ITGA2), Fms Related Tyrosine Kinase 4 (FLT1) and Vascular Endothelial Growth Factor B (VEGFB) were enriched in the local adhesion pathway (*P* ​= ​0.034) (Fig. 3B). Gene expression data showed that the relative expression of VEGFB186 (long transcript) and FLT1 were moderately higher in patients with AR compared with control subjects, while VEGFB167 (short transcript) and ITGA2 expression were significantly decreased in patients with AR compared with control subjects. (Figure S1).

Family 4 was further enlarged and the clinical and demographic data were shown in Supplementary E4. Sanger sequencing validated the rare variants in these 3 genes in family 1 and family 4 (Table 2 and Fig. 1). The c.603A ​> ​T substitution in exon 5 of FLT1 detected in family 1 results in a single amino acid substitution (glutamic acid to aspartic acid (p.E201D)) within Flt-1 domain 2 (Flt-1_D2_), which is important for VEGF binding.^30^ Furthermore, the c.603A ​> ​T in FLT1 was predicted to be functional by PolyPhen and PhyloP. Heterozygous variants c.322A ​> ​C; p.S108R in VEGFB and c.502+1G ​> ​A in ITGA2 were identified in Family 4; which interestingly exhibited intrafamilial heterogeneity. The father (Ⅱ8, proband), 1 of his children (Ⅲ8) and 2 of his relatives (Ⅱ3 and Ⅲ3) carried the c.322A ​> ​C in VEGFB, showing co-segregation of the rare variants with the AR phenotype. Similarly, the mother (Ⅱ9) carried another variant of c.502+1G ​> ​A in ITGA2, whereas a second child in the family (Ⅲ7) was a heterozygous carrier of both variants c.322A ​> ​C in VEGFB and c.502+1G ​> ​A in ITGA2. The second child (Ⅲ7) was found to have more severe clinical symptoms than the other single heterozygous carriers in this family. The c.322A ​> ​C substitution in VEGFB results in a single amino acid substitution (serine to arginine (p.S108R)) within the receptor-binding domain of VEGF (VEGF_8-109_), a homodimeric hormone that induces proliferation of endothelial cells.^30^ According to the 4 splice site prediction tools, the c.502+1G ​> ​A in ITGA2 was predicted to influence the formation of the splice donor site (Supplementary E5). The 2 rare variants were predicted to be functional by PolyPhen, PhyloP and Mutation taster.

**Table 2:** Mutations identified by Sanger sequencing in two families.

Sample	Gene	Position (hg19)	Mutation	Amino acid change	Functional prediction program				Allele frequence in ExAC^e^
					SIFT^a^	PolyPhen2^b^	PhyloP^c^	Mutation taster^d^	
Family1									
Mother	FLT1	Chr13:29008268	c.603A ​> ​T	p.E201D	Tolerated	Damaging	Conserved	Polymorphism	0.00127
Child	FLT1	Chr13:29008268	c.603A ​> ​T	p.E201D	Tolerated	Damaging	Conserved	Polymorphism	0.00127
Family4									
Ⅱ9 (Mother)	VEGFB	Chr11:64003747	c.322A ​> ​C	p. S108R	Tolerated	Damaging	Conserved	Disease causing	None
Ⅱ8 (Father)	ITGA2	Chr5:52344308	c.502+1G ​> ​A	Splice-site	–	–	–	Disease causing	0.0001161
Ⅲ7 (Child1)	VEGFB	Chr11:64003747	c.322A ​> ​C	p. S108R	Tolerated	Damaging	Conserved	Disease causing	None
	ITGA2	Chr5:52344308	c.502+1G ​> ​A	Splice-site	–	–	–	Disease causing	0.0001161
Ⅲ8 (Child2)	ITGA2	Chr5:52344308	c.502+1G ​> ​A	Splice-site	–	–	–	Disease causing	0.0001161
Ⅱ3	ITGA2	Chr5:52344308	c.502+1G ​> ​A	Splice-site	–	–	–	Disease causing	0.0001161
Ⅲ3	ITGA2	Chr5:52344308	c.502+1G ​> ​A	Splice-site	–	–	–	Disease causing	0.0001161

a SIFT, prediction of an amino acid change being damaging or tolerated.

b PolyPhen2, prediction of an amino acid change being damaging or benign.

c PhyloP, prediction of an amino acid substitution being conserved or non-conserved across species.

d Mutation taster, prediction of a disease-causing variant or polymorphism.

e Shown in East Asian population.

**Fig. 4:**
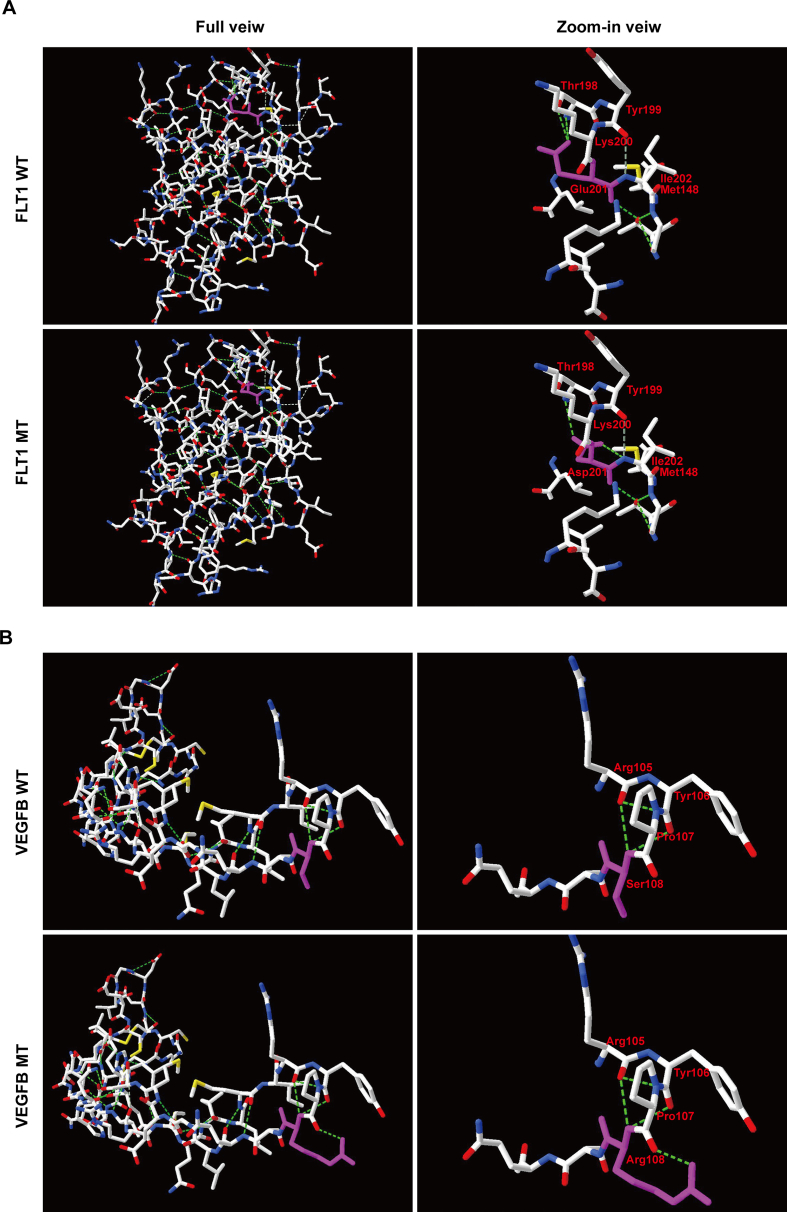
Structure of wild type and mutant type of p.E201D in FLT1 (A) and p. S108R in VEGFB (B), observed by Swiss-PdbViewer. Computed hydrogen bonds are shown as green dashed lines. Residues Glu201/Asp201 and Ser108/Arg108 are highlighted in pink.

To determine whether the identified 2 rare missense variants in FLT1 and VEGFB affect their binding, the 3D-structural models of FLT1 and VEGFB were predicted with SWISS-MODEL. The crystal structures of FLT1 (5t89 from the protein data bank, PDB) and VEGFB (2c7w1) were used as the templates, which was determined by X-ray diffraction at a resolution of 4 ​Å and 2.48 ​Å, respectively. The residues FLT1_Glu201 and VEGFB_Ser108 together with nearby residues within 6 ​Å, were illustrated. Flt-1_D2_ and VEGF_8-109_ were previously identified as the most important domains for ligand-receptor binding.^30^ As the Swiss-pdb Viewer 4.1 showing, at the resting state, FLT1_Glu201 forms H-bonds with Thr198 residue; when changed to Asp, the residue was predicted to lose an H-bond with Thr198 instead of to form a single H-bond with Ile202 residue. Similarly, when changed VEGFB_Ser108 into Arg, the residue was instead predicted to form a single H-bond with Pro107 residue. These mutations were predicted to perturb or change the amino acid side chains and would probably affect the hydrophobic interaction of Flt-1_D2_ with the “poles” of the VEGF dimer^30^ (Fig. 4).

Sanger sequencing showed that the 3 rare variants demonstrated in the 4 families were absent in both the groups of 96 sporadic HDM AR patients and 96 healthy control subjects. The demographic data were shown in Supplementary E6 and the specific oligonucleotide primers used for Sanger sequencing were shown in Supplementary E7.

## Discussion

This study identified 3 rare variants: in FLT1_c.603A ​> ​T, VEGFB_c.322A ​> ​C and ITGA2_c.502+1G ​> ​A, which may potentially be important in causing HDM AR. Moreover, the compound heterozygous carrier of c.322A ​> ​C in VEGFB and c.502+1G ​> ​A in ITGA2 had a more severe clinical phenotype. Indeed, VEGF and its receptors have previously been demonstrated to play an important role in nasal mucosal inflammation during AR^31^; thus taken together, these findings support the idea that the components of the local adhesion pathway are likely to be implicated in HDM-induced AR.

A family history of an allergic condition is a well-accepted risk factor for the development of an allergic condition in an individual, particularly for allergic disorders such as asthma, eczema, and AR. However, the question of whether specific allergen sensitization is inherited has been considered to be a complicated matter. Misiak et al.^32^ reviewed the literature regarding this issue and concluded that specific allergen sensitization was influenced by both a genetic component and environmental exposure to allergen – with no clear-cut answer despite accounting for not only the complexity of allergy as a trait itself but also for the complexities of varying study methodologies as well as the evaluation of diverse populations and communities.

Candidate gene association studies and GWASs have demonstrated the presence of susceptibility loci, which conferred relatively small increments in risk but not the full profile of the genetic characteristics of allergy, thus suggesting restricted ability of these techniques to find rare risk alleles which might contribute to the disease.^33^ The next generation sequencing (NGS) techniques have led to a new approach to identify new causal genes and mutations in monogenic as well as complex diseases.^34^ In the present study, the filtering strategy combining both the whole genome sequencing data and the existing AR related gene expression profiling data is rather conservative. Given that the largest proportion of common variants are not associated with disease, we chose to focus our attention on variants not present in variant databases such as dbSNP143 and 1000 Genomes Project. Moreover, here we focused on variants that might only be identified through the use of a family-based design, which has been successfully employed to identify asthma susceptibility variants.^35^ However, this study of asthma investigated only 1 family segregating asthma by applying exome sequencing and reported several potentially functional variants in potential asthma candidate genes.^35^ In the present study, we have investigated 4 families segregating DM-induced AR by genome-wide sequencing and combined the data from a previous global gene expression study of nasal mucosa from 7 AR patients and 5 non-allergic control subjects to analyze distinct gene expression within the whole genome sequencing data. This technique identified 3 rare variants in 3 genes (FLT1_c.603A ​> ​T, VEGFB_c.322A ​> ​C, ITGA2_c.502+1G ​> ​A; involved in Focal Adhesion pathway), which were present in affected subjects in 2 families but were absent in unaffected individuals. Although the large consortium-based GWAS is likely sufficiently powered to identify associations with even rare variants,^36^ it may be that the 3 variants detected in the present study are not only rare but also segregated with DM AR in only some families. In this sense, it seems plausible that we failed to detect these 3 variants in the sporadic DM AR patients and the healthy control subjects, particularly as the sample size was too small. Indeed, a very recently published study has indicated that searching for rare coding or splice site variants in very large sample sizes can help prioritize causal genes at many GWAS loci associated with complex human diseases and traits.^37^ Thus, it is possible that a further replication study in a large cohort of HD AR and healthy subjects may confirm the findings for the 3 rare variants in the present study in the future.

Assessment of increased numbers of individuals in Family 4, exhibited the presence of intrafamilial heterogeneity and heterozygous variants c.322A ​> ​C; p.S108R in VEGFB and c.502+1G ​> ​A in ITGA2. It is noteworthy that all the DM AR patients in this family carried either 1 or both of the variants and that the compound heterozygous carrier of both these variants had more severe clinical symptoms and increased serum DM levels. We thus hypothesised that there may exist family-specific mutations, and, further, at least 1 of these mutations was necessary to lead to the development of AR, and in combination the mutations would additionally control the severity of the disease. This could be in accordance with the theory that certain combinations of susceptibility genes are specific for allergic airways disease and/or are associated with severity of disease.^38^

Increased vessel number and permeability are important features of the nasal mucosa in AR, and are mediated in part by the cytokine VEGF, a major regulator of angiogenesis and enhancer of vascular permeability. In mammals, the VEGF family consists of VEGFA, VEGFB, VEGFC, VEGFD, and placenta growth factor (PLGF), with each member having a specific site of expression and showing distinct binding pattern to the different VEGF receptors (VEGFRs).^39^ The biological effects of VEGF are mediated by 3 VEGFRs; including VEGFR-1 (FLT 1), VEGFR-2 (KDR/FLK-1), and VEGFR-3 (FLT 4)^31^; of which VEGFR-1 is a positive regulator of monocyte and macrophage migration and a positive and negative regulator of VEGFR-2 signaling capacity and bind VEGFA, VEGFB, and PLGF.^39^VEGF levels have been shown to be increased in the nasal mucosa of patients with AR^40^ and contribute to nasal inflammation in response to allergen exposure.^41^More recently, Chen et al.^42^ demonstrated that DM extract induced the expression of VEGF, transforming growth factor-β1 (TGF-β1), and fibroblast growth factor-2 (FGF-2) by activating the PI3K/Akt/HIF-1a pathway in human primary cultured nasal epithelia cells (NECs) and in the nasal mucosa of a murine model.^42^ Furthermore, the demonstration that the expression of VEGFR1 and VEGF-B correlates with edema and clinical markers of nasal polyps (NP) disease is noteworthy, particularly as edema represents a key feature of NP disease, and therefore represents potential therapeutic targets.^43^ Little is known about the roles of ITGA2 in the pathogenesis in AR. ITGA2 encodes for integrin, playing important functions in cell adhesion, angiogenesis and innate and acquired immune response.^44^ One recent study investigated genome-wide expression of microRNAs with total and translational mRNA in human primary bronchoepithelium from severe asthmatics and healthy controls.^45^ The authors demonstrated that the skipped isoforms of ITGA2 presented increased polyribosome binding in severe asthma. Collectively, these findings and the findings from the present study suggest that the further functional study on VEGFs and their receptors and ITGA2 may shed meaningful light on the genetic mechanisms and potential therapeutic targets relevant to AR and the related diseases.

Last but not least, even though the greatest effect of studies on genetics of AR has been in increasing our understanding of disease pathogenesis, there are still ways in which the findings regarding genetic basis of AR will improve diagnosis and treatment in the future. To begin with, identification of mutations in the 3 genes can enable more accurate prediction of the likelihood that a subject will develop DM AR and that facilitating lifestyle change to avoid DM allergen exposure will be valuable. Secondly, it has been convincing that genetics might play an important role in prediction is in disease severity, and the ability to identify those who are most likely to have severe persistent disease would allow targeting of preventative treatments and be of significant clinical utility. For example, here we reported that the compound heterozygous carrier of both c.322A ​> ​C; p.S108R in VEGFB and c.502+1G ​> ​A in ITGA2 had more severe clinical symptoms and serum DM levels; thus, more attention should be exerted towards such population. In addition, the further investigation concerning the relationship between asthma phenotype and the identified mutations would shed light on the issue regarding how to predict the potential asthma subtypes in DM AR subjects.

In summary, our findings suggest that 3 rare variants in FLT1_c.603A ​> ​T, VEGFB_c.322A ​> ​C and ITGA2_c.502+1G ​> ​A are probably causal variants in DM AR. Although allergy is widely considered to be a complex disorder, our study further suggests that these variants are present within several families, indicating a strong involvement of the genetic components. However, further studies replicating the methodologies employed in present study in a much larger population as well as functional studies are required to determine the relationship among the rare variants found in the 3 genes in patients with AR.

## Conclusions

Three rare variants in 3 genes (FLT1_c.603A ​> ​T; VEGFB_c.322A ​> ​C; and ITGA2_c.502+1G ​> ​A), which are involved in Focal Adhesion pathway, were identified in affected AR subjects in DM AR families by whole-genome sequencing and conjoint analysis of gene expression profiling data. Despite the relatively small sample size, this study has identified several potentially functional rare variants in AR candidate genes, and it provides a cutting edge opportunity for future work in larger numbers of families and sporadic individuals for a better understanding of the genetic basis of AR.

## Declarations

### Ethics approval and consent to participate

The ethics review board of the Beijing Tongren hospital, P.R. China, approved the study and, prior to entry into the study, all participants provided written informed consent.

### Consent for publication

Not applicable.

### Availability of data and materials

The datasets generated during the current study are available from the corresponding author on reasonable request.

### Competing interests

All authors declare that they have no competing interests.

## Fundings

This work was supported by grants from the national key R&D program of China (2016YFC0905200), the program for the Changjiang scholars and innovative research team (IRT13082), the national natural science foundation of China (81420108009, 81630023, 81570895, 8140044 and 481400447), Beijing Natural Science Foundation (7182034), Beijing municipal administration of hospitals' mission plan (SML20150203), and Beijing advanced innovation center for food nutrition and human health (Beijing Technology and Business University, 20181045), Beijing Health Bureau Program for High Level Talents (2014-3-018) and Cross-disciplinary Collaborative Program of Beijing Nova program (xxjc201712).

### Authors' contributions

All the authors contributed significantly to the study: YZ, JL and YZ collected the data. JL performed statistical analyses. YZ and JL wrote the manuscript. CW and LZ designed and supervised the study.

## Figures and Tables

**Figs1:**
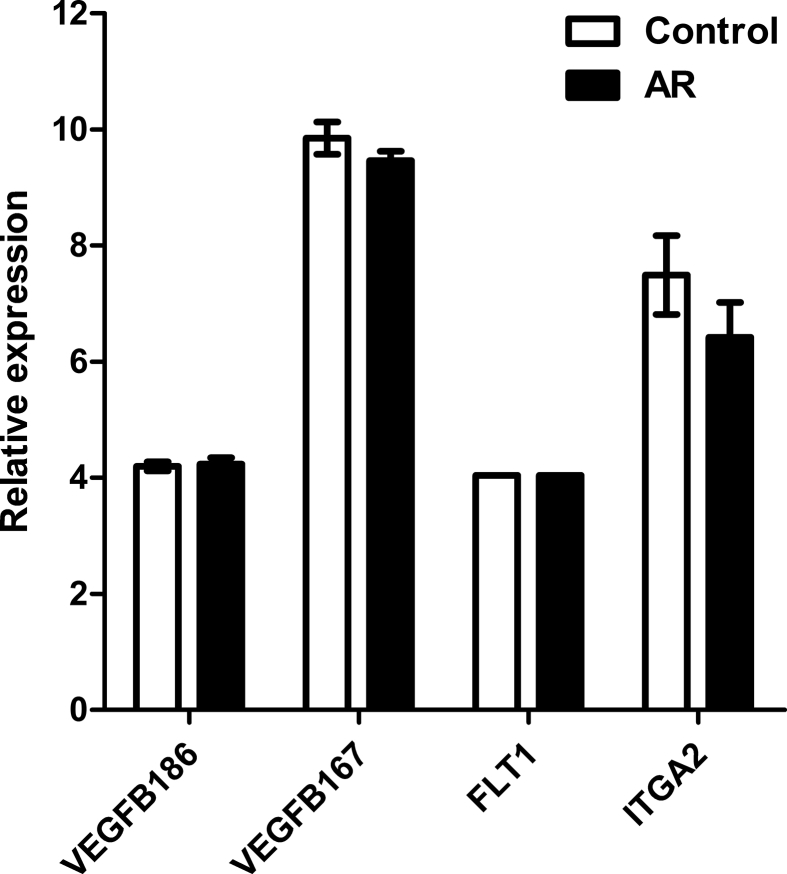

