# Supplementary material for: Identification of rare variants of allergic rhinitis based on whole genome sequencing and gene expression profiling: A preliminary investigation in four families
Source: World Allergy Organ J. 2019 Jun 13;12(6):100038. doi: 10.1016/j.waojou.2019.100038 (PMC6581771; doi:10.1016/j.waojou.2019.100038)
Supplement: Multimedia component 1 [file mmc1.doc]

**Supplementary tables：**

**Supplementary E1**. Clinical and Demographic information in four AR families for whole genome sequencing.

| Family number | Family member | Gender | Age  （Years） | AR diagnosis | Allergens | Other allergic diseases |
| --- | --- | --- | --- | --- | --- | --- |
| 1 | Mother | Female | 38 | Yes | HDM | No |
|  | Father | Male | 40 | No | - | No |
|  | Child | Female | 14 | Yes | HDM | No |
| 2 | Mother | Female | 45 | Yes | HDM | No |
|  | Father | Male | 47 | No | - | No |
|  | Child | Female | 19 | Yes | HDM | No |
| 3 | Mother | Female | 37 | Yes | HDM | No |
|  | Father | Male | 38 | No | - | No |
|  | Child | Male | 9 | Yes | HDM | No |
| 4 | Mother | Female | 57 | Yes | HDM | No |
|  | Father | Male | 57 | Yes | HDM | No |
|  | Child1 | Female | 29 | Yes | HDM | No |

**Supplementary E2**. Summary for whole genome sequencing data of nine AR patients in four families

| Family number | Family member | Bases（G） | Map Bases | Total reads | Exon coverage | Genome coverage | Sequencing depth | SNVs | Indels |
| --- | --- | --- | --- | --- | --- | --- | --- | --- | --- |
| 1 | Mother | 50.86 | 50,053,469,203 | 495,578,903 | 82.27% | 91.65% | 13X | 3,200,356 | 355,345 |
|  | Child | 60.54 | 59,476,955,346 | 588,880,746 | 81.63% | 91.62% | 16X | 3,295,274 | 426,310 |
| 2 | Mother | 70.83 | 68,294,856,800 | 682,948,568 | 91.79% | 91.67% | 22X | 2,854,743 | 260,350 |
|  | Child | 42.77 | 42,286,535,974 | 418,678,574 | 79.05% | 91.32% | 11X | 3,447,537 | 547,838 |
| 3 | Mother | 52.52 | 51,806,492,469 | 512,935,569 | 89.14% | 91.60% | 15X | 3,414,558 | 424,263 |
|  | Child | 60.22 | 59,645,690,100 | 596,456,901 | 86.57% | 92.30% | 17X | 3,380,941 | 391,551 |
| 4 | Mother | 48.89 | 47,971,477,726 | 474,965,126 | 91.61% | 92.29% | 15X | 3,418,721 | 411,384 |
|  | Father | 44.09 | 42,828,959,706 | 424,049,106 | 92.02% | 91.61% | 13X | 3,453,289 | 447,227 |
|  | Child1 | 44.95 | 44,102,746,052 | 436,660,852 | 92.18% | 91.66% | 13X | 3,442,478 | 412,398 |

SNVs, sigle nucleotide variants; Indels, insertions/deletions

**Supplementary E3.** Family shared NS/SS/I detected by whole genome sequencing.

| Gene | Position (hg19) | Mutation | Amino acid change | Allele frequence in ExAC^*^ | Family shared |
| --- | --- | --- | --- | --- | --- |
| KCNG4 | Chr16:84256452 | c.931 G>A | p.A311T | None | Family 1, Family 4 |
| NCOA6 | Chr20:33345504  Chr20:33328547 | c.1047 G>C  c.5513 C>G | p.L349F  p.S1838C | 0.0004622  None | Family 1, Family 4 |
| KIAA1217 | Chr10:24832431  Chr10:24820834 | c.4232 T>C  c.3158 G>A | p.I1411T  p.R1053H | None  0.0001159 | Family 2, Family 3 |

^*^Shown in East Asian population.

NS, nonsynonymous variant; SS, splice-site acceptor or donor variants; I, coding insertions or deletions.

**Supplementary E4.** Clinical and Demographic findings for members of family number 4.

| No. | Gender | Age (Years) | AR diagnosis | Allergens | Other allergic diseases |
| --- | --- | --- | --- | --- | --- |
| Ⅱ3 | Female | 59 | Yes | HDM | No |
| Ⅱ4 | Male | 59 | No | No | No |
| Ⅱ5 | Female | 55 | No | No | No |
| Ⅱ6 | Male | 53 | No | No | No |
| Ⅱ8 | Male | 57 | Yes | HDM | No |
| Ⅱ9 | Female | 57 | Yes | HDM | No |
| Ⅱ10 | Female | 65 | No | No | No |
| Ⅲ1 | Male | 46 | No | No | No |
| Ⅲ2 | Female | 47 | No | No | No |
| Ⅲ3 | Male | 34 | Yes | HDM | No |
| Ⅲ4 | Male | 32 | No | No | No |
| Ⅲ5 | Male | 32 | No | No | No |
| Ⅲ6 | Male | 29 | No | No | No |
| Ⅲ7 | Female | 29 | Yes | HDM | No |
| Ⅲ8 | Male | 24 | Yes | HDM | No |
| Ⅳ1 | Male | 16 | No | No | No |
| Ⅳ3 | Male | 28 | No | No | No |

**Supplementary E5**. Results by Splicing Prediction Programs for c.502+1G>A in ITGA2.

| Program | Human Splicing Finder | MaxEntScan | Spliceman | NetGene2 |
| --- | --- | --- | --- | --- |
| Scores | Consensus value for donor site^1^ | Score for 5’（donor）site^2^ | Percentile Rank^3^ | Neuronal network donor site score^4^ |
|  | WT= 83.18  MT= 56.34  Variation (%) = -32.27 | WT= 10.07  MT= 1.89  Variation (%) = -81.23 | 68% | WT= 0.990  MT= 0.0 |

^1^ The Consensus value for donor site is caculated based on the algorithm of Position Weight Matrices. If WT value > 65 and the variation (%) < -10%, the mutation breaks the splice site.

^2^ The Score for 5’（donor）site is caculated based on the algorithm of Maximum Entropy Model. If WT score > 3 and the variation (%) < -30%, the mutation breaks the splice site.

^3^ The program uses the Computing distance matrix which is caculated to qualify the “closeness” between two hexamers. The hexamer most likely prone t`o splicing is selected. The values of distance are converted into percentile rank. The Higher the percentile rank, the more likely the point mutation is to disrupt splicing.

^4^ The donor site score is calculated based on the the neuronal network method. The output of the network is a score between 0 and 1 for a potential splice site. The score with a hight close to 1.0 indicates a strong donor site.

WT, Wild Type; MT, Mutant Type.

**Supplementary E6.** Demographic characteristics of individuals with sporadic AR and Control subjects.

|  | AR | Control |
| --- | --- | --- |
| No. | 96 | 96 |
| Gender (male/female) | 50/46 | 54/42 |
| Age, mean (SD) | 33 (10) | 35 (14) |

**Supplementary E7.** Specific oligonucleotide primers used for Sanger Sequencing.

| Gene | Forward primer Sequence 5' -> 3' | Reverse primer Sequence 5' -> 3' | Product (bp) |
| --- | --- | --- | --- |
| FLT1 | GGTCTATCCTGGGTAGATGC | CGTGTTCAAGGGAGTGGTA | 648 |
| VEGFB | CCAGGTGTCCATCATCTTGTG | TTCTGTGGACCACCATACCC | 839 |
| ITGA2 | CTCCCTTCACTGACACCT | CAGTGAGCCTAAATCGTG | 982 |
